# Co-administration of xylo-oligosaccharides produced by immobilized *Aspergillus terreus* xylanase with carbimazole to mitigate its adverse effects on the adrenal gland

**DOI:** 10.1038/s41598-024-67310-4

**Published:** 2024-07-30

**Authors:** Shaimaa A. Nour, Doaa S. Foda, Islam A. Elsehemy, Mohamed E. Hassan

**Affiliations:** 1 Chemistry of Natural and Microbial Products Department, Pharmaceutical and Drug Industries Research Institute, National Research Centre, El Behouth Street, Cairo, 12622 Egypt; 2 Therapeutic Chemistry Department, Pharmaceutical and Drug Industries Research Institute, National Research Centre, El Behouth Street, Cairo, 12622 Egypt; 3 Centre of Excellence, Encapsulation and Nano Biotechnology Group, Chemistry of Natural and Microbial Products Department, Pharmaceutical and Drug Industries Research Institute, National Research Centre, El Behouth Street, Cairo, 12622 Egypt

**Keywords:** Immobilized xylanase, Microbial scleroglucan, Covalent immobilization, Xylo-oligosaccharides, Carbimazole drug, Adrenal glands, Biological techniques, Biotechnology

## Abstract

Carbimazole has disadvantages on different body organs, especially the thyroid gland and, rarely, the adrenal glands. Most studies have not suggested any solution or medication for ameliorating the noxious effects of drugs on the glands. Our study focused on the production of xylooligosaccharide (XOS), which, when coadministered with carbimazole, relieves the toxic effects of the drug on the adrenal glands. In addition to accelerating the regeneration of adrenal gland cells, XOS significantly decreases the oxidative stress caused by obesity. This XOS produced by *Aspergillus terreus* xylanase was covalently immobilized using microbial Scleroglucan gel beads, which improved the immobilization yield, efficiency, and operational stability. Over a wide pH range (6–7.5), the covalent immobilization of xylanase on scleroglucan increased xylanase activity compared to that of its free form. Additionally, the reaction temperature was increased to 65 °C. However, the immobilized enzyme demonstrated superior thermal stability, sustaining 80.22% of its original activity at 60 °C for 120 min. Additionally, the full activity of the immobilized enzyme was sustained after 12 consecutive cycles, and the activity reached 78.33% after 18 cycles. After 41 days of storage at 4 °C, the immobilized enzyme was still active at approximately 98%. The immobilized enzyme has the capability to produce xylo-oligosaccharides (XOSs). Subsequently, these XOSs can be coadministered alongside carbimazole to mitigate the adverse effects of the drug on the adrenal glands. In addition to accelerating the regeneration of adrenal gland cells, XOS significantly decreases the oxidative stress caused by obesity.

## Introduction

One of the most important enzymes that can be used in xylan diodegradation is xylanase (1,4-beta-D-xylan xylanohydrolase, EC 3.2.1.8)^1^. Compared to traditional synthesis methods, enzyme-mediated catalytic processes have several advantages in the biotechnological and industrial fields^2^. Enzymes are typically handicapped and limited by constraints such as no recovery, high cost, short catalytic lifetime, nonrecyclability, and poor operational stability against pH, temperature, and other factors^3^. A potential solution to these problems is the technique of immobilizing enzymes on appropriate carrier supports, which increases the stability and activity of the immobilized enzyme compared with those of the free enzyme. Improving the characteristics and reusability of enzymes is another objective of enzyme immobilization^2^.

The operational stability of enzymes may be improved by immobilizing them on a solid matrix. Additionally, because the enzyme is immobilized on an inert carrier, it is possible to reuse the enzyme in several reactions and product separation cycles, which results in significant cost savings^4^. Methods of enzyme immobilization, such as entrapment, adsorption on solid carriers, and ionic or covalent binding, are often utilized to immobilize enzymes^5^.

The immobilization of enzymes can enhance their stability, efficiency, reusability, and cost-effectiveness. Stability is improved through techniques such as covalent immobilization, which prevents subunit dissociation, and strategies aimed at preventing enzyme aggregation and degradation. Multipoint covalent attachment is an additional technique that enhances the structural integrity of the enzyme. In addition to being stable, immobilization creates a favorable environment for enzyme activity, although it may also lead to decreased enzyme activity. Factors such as partial blocking of active sites, limitations in mass transfer, and conformational changes can hinder catalysis. Therefore, there is a strong need for a new immobilization method that can simultaneously enhance enzyme activity and stability^6,7^.

Enzyme immobilization has been extensively employed to enhance enzyme catalytic properties, inhibit denaturation, and improve economic viability for various applications. There are many benefits to using immobilization to improve the stability and reusability of enzymes. Enzyme immobilization has been studied for a variety of materials, including metal oxides, acrylic polymers, silica, and carbons^8^. In numerous biomedical applications, including cell culture, drug delivery, tissue engineering, and the immobilization of metals or enzymes for use as catalysts, alginate—a kind of polysaccharide—is frequently employed as a biopolymer. Because of its good biocompatibility, soft reducibility, and affordability, alginate has emerged as the preferred carrier in immobilization technology^9,10^.

Among different immobilization techniques, the covalent method is often preferred. This is because it creates a strong covalent bond between the functional group of the enzyme and the functional group of the carrier. This type of binding ensures that the enzyme stays securely immobilized on the carrier surface, which leads to optimal activity when the immobilized enzyme is reused in consecutive cycles. Additionally, the multipoint-covalent linkages between the carrier and enzyme molecules provide protection against unfolding and denaturation. Covalent binding is widely used in various industrial enzyme applications because it helps to reduce enzyme leakage by strengthening the bond between the carrier and the enzyme^11,12^.

Alginate has been employed in immobilization technology by covalent methods^13^ or entrapment^14^. This can be achieved by activation with glutaraldehyde (GA) after reaction with polyethyleneimine (PEI)^15^. An efficient complex of alginate/scleroglucan beads was created by adding scleroglucan to alginate to boost its mechanical strength and immobilization effectiveness.

The main producers of scleroglucan, a natural exopolysaccharide, are members of the genus *Sclerotium*. It is composed of a D-(1–3)-glucopyranosyl main linear chain with a branch connecting a D-glucan unit (1–6) to every third unit (Fig. S1).

In particular, for the development of certain products and methods, scleroglucan stands out from other natural polysaccharides due to its variety of distinctive chemical and physical properties (such as its resistance to temperature, hydrolysis, and electrolytes). In addition, these materials might find use in the pharmaceutical industry, the food industry, medicinal delivery systems, and the cosmetic industry. Scleroglucan performs a wide range of mechanical tasks, mostly in the oil industry, where it is used to thicken, penetrate mud, improve oil recovery, and prepare adhesives, print inks, and animal feed. All of these uses are attributed to the unique rheological characteristics of these materials^16^.

The carrier was developed through the improvement of the mechanical characteristics of alginate via the incorporation of scleroglucan, resulting in an increased number of active groups on the surface of the alginate/scleroglucan complex. The utilization of this complex in the immobilization process involves the formation of covalent bonds with the enzyme. By enabling multiple reuses for enzyme immobilization, this novel carrier effectively mitigates the associated costs. Furthermore, the obtained results exhibit noteworthy enzyme activity, rendering them suitable for a large number of uses in both the medical and industrial sectors^17^.

Additionally, *Aspergillus terreus* xylanase is an excellent xylanase producer and has the capacity to manufacture large amounts of xylanase^18^. To make xylooligosaccharides from the xylan of beech wood, xylanase is used. Applying immobilized enzymes in this procedure will make it possible to reuse the xylanases and simplify the procedure.

Among the most vital organs of the human and animal endocrine systems is the adrenal gland^19^. It is located near the anterior poles of the kidneys^20^. The glands secrete different important hormones that are related to controlling body homeostasis^21^. Adrenal gland hormones directly affect each important organ in humans, especially the heart, nervous system, kidneys, muscles, and bones. This effect also includes the process by which lipids, proteins, and carbohydrates undergo breakdown^22^.

The gland is divided into two main sections: the outer layer, called the cortex, is composed of three layers: the zona glomerulosa, which is the outermost layer; the zona reticularis, which is the intermediate layer; and the medulla, which is the inner layer, which consists mainly of chromaffin cells^23^. The adrenal glands are strongly affected by xenobiotics, which can result in many pathological conditions^24,25^.

In this study, we propose utilizing xylo-oligosaccharides (XOSs) derived from xylan degradation by *Aspergillus terreus* xylanase to mitigate the harmful effects of the antithyroid medication carbimazole on the structural integrity of the adrenal gland. This finding suggests a new application of XOS for this specific purpose.

This study’s primary goals are to assess *Aspergillus terreus* xylanase immobilization on microbial scleroglucan and apply it to the direct hydrolysis of xylan in beech wood. The goal of this study was to increase the yield of xylooligosaccharides and explore their potential use as in vivo detoxifying agents.

## Materials and methods

### Materials

Alginate sodium salt (alg.) was purchased from Fluka. Scleroglucan was prepared in our laboratory. Glutaraldehyde (GA), polyethyleneimine (PEI), and beech wood xylan polymer were purchased from Sigma‒Aldrich, Germany. 3,5-Dinitrosalicylic acid (DNS) was purchased from Pan Reac, Barcelona, Spain. The other chemicals used were of Analar grade or equivalent quality.

### Methods

#### Production of* Aspergillus terreus* xylanase

The xylanase used in this investigation was generated using the techniques of Nour et al. This process involved solid-state fermentation of *Ricinus communis* waste using *Aspergillus terreus* RGS Eg-NRC (accession number MW282328)^18^.

The media consisted of the following (g/L): *Ricinus communis*, 20 g; corn steep, 10 g; KH_2_PO_4_, 6.5 g; glucose, 40 g; and the moistening agent (60 mL/L). The media were subsequently incubated for 11 days at 30 °C. The fermented substrate was centrifuged at 5000 rpm for 10 min at 4 °C after extraction with 50 mL of distilled water after being incubated on an orbital shaker (150 rpm) for 1 h. The resulting supernatant was subjected to enzyme analysis^18^.

Xylanase, which was prepared in our laboratory, was used to enzymatically hydrolyze the xylan polymer found in beech wood to create XOS.

#### Estimation of the enzyme, protein and purification of xylanase

Then, 0.5 mL of diluted xylanase extract was mixed with 0.5 mL of beach wood xylan solution (1%) in 50 mM phosphate buffer (pH 6) for 30 min at 50 °C. The enzyme activity was ascertained according to Bailey et al.^26^ DNS (2.5 mL) was added to halt the reaction once it cooled. The emission of the sugar xylose was recorded at 540 nm. One unit (U) of xylanase activity releases one mole of crude extract of enzyme per milliliter of xylose.

The amount of protein was calculated using bovine serum albumin (BSA) in accordance with the methods of Lowry et al*.*^27^ Following fractional precipitation of enzymes using ammonium sulfate, a fraction of ammonium sulfate (60–70%) was utilized for xylan hydrolysis^18^.

#### Carrier preparation for covalent immobilization (alginate/scleroglucangel beads)

Alginate/scleroglucan (Alg/Sc.) Gel beads were created by utilizing an overhead mechanical stirrer to dissolve alginate (Alg) and scleroglucan (Sc) in distilled water at a 1:1 ratio to a final concentration of 2%. The compound was injected into 3% CaCl_2_ with a 300 mm nozzle utilizing an Innotech Encapsulator (IE-50, Switzerland).

#### Activation of gel beads

**Figure 1:**
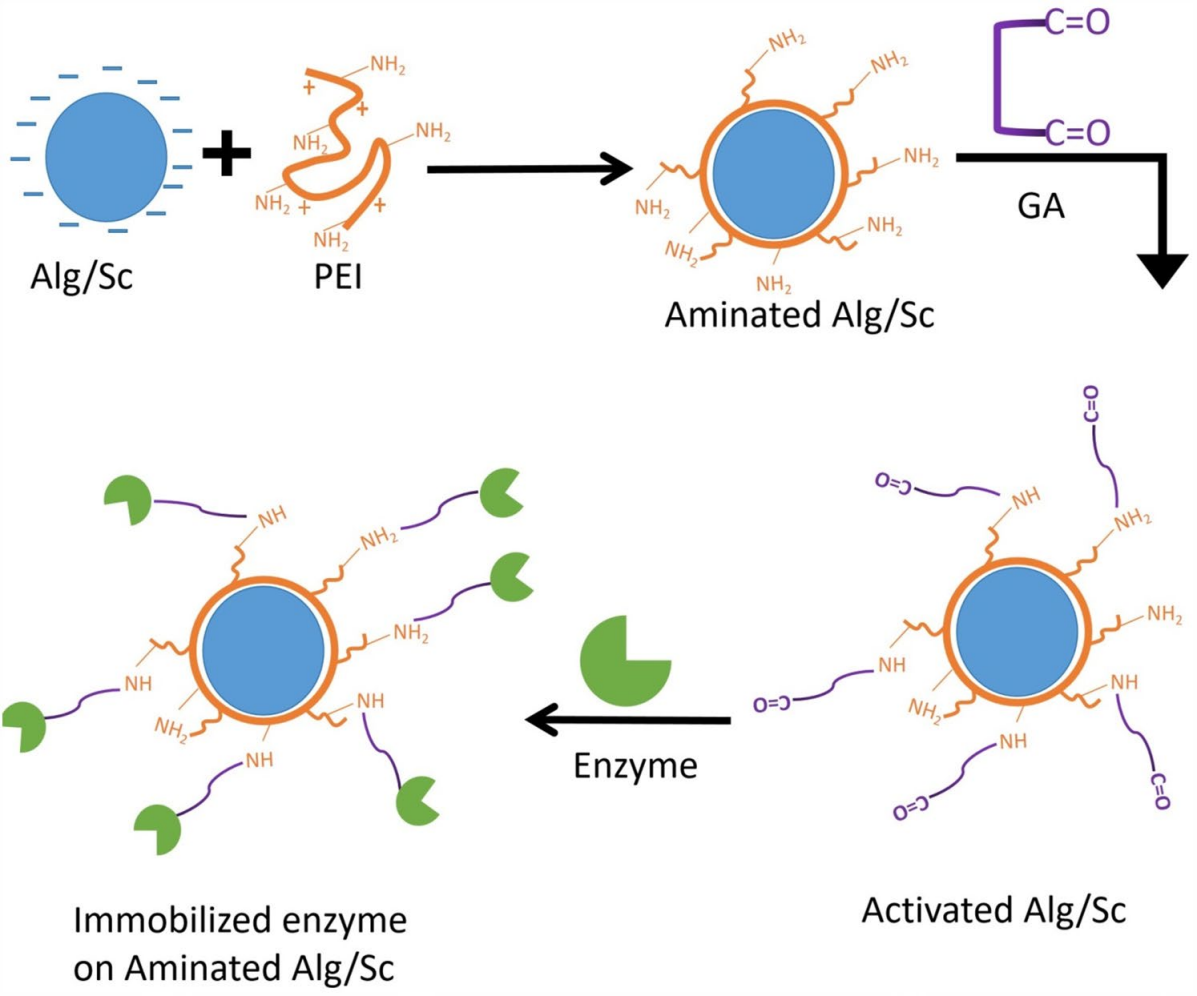
Activation scheme and immobilization processes of Alginat/Scleroglucangel beads.

To prepare Alg/Sc. beads to immobilize covalent bonds, activation using polyethyleneimine (PEI) followed by glutaraldehyde (GA) can be used. Alg/Sc. The beads were first presoaked for three hours in 4% PEI at pH 9.5 before being thoroughly rinsed with distilled H_2_O to remove any remaining PEI. After that, the aminated Alg/Sc beads were submerged for three hours in a 2.5% GA solution. Then, three rinses were performed on the unreacted GA from the Alg/Sc. beads, as depicted in Fig. 1. The activated beads could now be employed in the subsequent stages of enzyme immobilization^28,29^.

#### Xylanase immobilization on activated alginate/scleroglucan

The xylanase enzyme was immobilized on alginate/scleroglucan (Alg/Sc.) After being steeped in a 2.5 U enzyme solution, the specimens were incubated at room temperature for a whole day. To remove any remaining enzyme following immobilization, the beads were rinsed three times with distilled water. The cleaned beads were stored at 4 °C in phosphate buffer (pH 5.6) for further measurements. The immobilization yield was calculated as follows:

1 $${\text{Immobilization Yield }}\left( {\text{\% }} \right) = {\text{I}}/\left( {{\text{A}} - {\text{B}}} \right)$$

I is the amount of immobilized enzyme (U/carrier), A is the total amount of enzyme added (U/carrier), and B is the amount of unbound enzyme (U/carrier). I is the quantity of enzyme that was immobilized (U/carrier), A is the total quantity of enzyme added (U/carrier), and B is the amount of unattached enzyme (U/carrier).

#### Statistical optimization of the immobilization process

By modifying Box and Wilson^30^, a central composite design (CCD) was implemented. To identify the factors that are independent of loading time (X1) and enzyme units added (X2), thirteen trials were carried out. The link between the independent components and the immobilization yield was ascertained using a second-order polynomial function:

2 $$Y = \beta_{0} + \beta_{1} X_{1} + \beta_{2} X_{2} + \beta_{11} X_{1}^{2} + \beta_{22} X_{2}^{2} + \beta_{1} \beta_{2} X_{1} X_{2}$$

where Y is the immobilization yield (%), β_0_ is the intercept, β_1_ and β_2_ are linear coefficients, β_11_ and β_22_ are quadratic coefficients, and β_12_ is the cross-product coefficient.

#### Characterization of the modified gel beads using FT-IR

An FT-IR spectrophotometer was used to obtain the infrared transmission spectra of alginate/scleroglucan, alginate/scleroglucan/PEI, alginate/scleroglucan/PEI/GA, and alginate/scleroglucan/PEI/GA/Enzyme (FT-IR-800, Shimadzu, Japan). This process is employed to demonstrate how gel beads change as a result of the presence of the correct functional groups. Before the four formulations were mixed with KBr, each sample was allowed to air dry. After being crushed into an extremely fine powder in a mortar, the discs were compacted into discs using a hydraulic press operating at 10,000 pounds per square inch. The samples were scanned in the 400–4000 cm^−1^ wavelength range at ambient temperature^28^.

#### Immobilized xylanase characteristics

##### Temperature and pH effects

The impact of pH on enzyme activity between pH values of 5.7 and 9 was investigated. Two buffers were used: a 0.05 M phosphate buffer with pH values between 5.7 and 8.0 and a Tris HCl buffer with pH values between 8.5 and 9.0. At the optimal pH, the enzyme functioned at its maximum. Additionally, the stability of the enzyme over a range of pH values was established. The remaining activity was measured every 30 min for up to two hours following preincubation at every pH. Without prior incubation, the enzyme was regarded as 100% active.

The impact of temperature on the activity of the immobilized enzyme was evaluated at different temperatures (40–80 °C) and at the optimal pH. The activation energy (Ea) of the generated enzyme was determined by plotting the relative activity against the reciprocal of temperature in Kelvin, or the Arrhenius plot, using the following equation:

3 $$Slope = E_{a} /R$$

R, the gas constant.

##### Temperature stability

The residual activity of xylanase after being immobilized under ideal conditions was estimated every 30 min for up to two hours after preincubation at 40–75 °C. When the previous incubation did not occur, the enzyme activity was evaluated as 100%.

##### Effect of different substrate concentrations

We estimated the *Aspergillus terreus* xylanase activity using different concentrations of xylan (2.5–25 mg/ml) at the optimum pH and temperature of the tested enzymes. From the Lineweaver–Burk plot equation (Eq. 4), we calculated the maximum reaction velocity (V_*max*_), Michaelis–Menten constant (K_*m*_), and specificity constant (V_*max*_/K_*m*_). The reaction velocity (V) is represented as the specific activity in U/mg protein, and S is the concentration of xylan (mg/mL).

4 $${\raise0.7ex\hbox{$1$} \!\mathord{\left/ {\vphantom {1 V}}\right.\kern-0pt} \!\lower0.7ex\hbox{$V$}} = \left( {{\raise0.7ex\hbox{$1$} \!\mathord{\left/ {\vphantom {1 {Vmax}}}\right.\kern-0pt} \!\lower0.7ex\hbox{${Vmax}$}}} \right) + \left( {{\raise0.7ex\hbox{${Km}$} \!\mathord{\left/ {\vphantom {{Km} {Vmax}}}\right.\kern-0pt} \!\lower0.7ex\hbox{${Vmax}$}}} \right)\left( {{\raise0.7ex\hbox{$1$} \!\mathord{\left/ {\vphantom {1 S}}\right.\kern-0pt} \!\lower0.7ex\hbox{$S$}}} \right)$$

#### Enzyme reusability

Under ideal circumstances, it was determined whether the immobilized enzyme could hydrolyze xylan in beech wood. When the reaction was complete, the beads were removed and washed before being added to another reaction. As previously mentioned, the amount of freed xylose in the supernatant was calculated after every reaction. The beads were stored at 4 °C while immersed in phosphate buffer, after which the activity of the immobilized enzyme was monitored weekly for one month to determine the enzyme storage stability.

#### Xylan hydrolysis for xylooligosaccharide production

To test the activity of the immobilized enzyme in hydrolyzing beech wood xylan, 0.5 mL of 1% xylan in 0.05 M phosphate buffer (pH 5.8) containing 1 g of beads (2.65 U) was suspended in the mixture. The reaction mixture was incubated for one hour at 120 rpm and 40 °C. At the conclusion of the hydrolysis process, the beads were removed from the mixture. After that, the liquid was boiled for ten minutes to denature the additional enzyme, which halted the processes. Ultimately, the mixture was centrifuged for ten minutes at 4 °C to remove the remaining unhydrolyzed substrate at 7000 rpm. The reducing sugar content in the supernatant was measured using the DNS technique^31^.

#### Chromatography using thin layers

Using silica gel plates, TLC was used to separate and identify the carbohydrates. The samples were spread out across plates containing various real sugars (mono, di, tri, and tetra). Plates were made in a saturated chamber at ambient temperature using propanol:water (8.5:1.5 v/v) as the mobile phase. An approach involving spraying was used to identify sugars. The dried plates were then sprayed with phenol‒sulfuric acid reagent (3 g of phenol and 5 mL of highly concentrated sulfuric acid in 95 mL of ethyl alcohol), and the mixture was incubated for ten to fifteen minutes at 100 °C^32^.

#### In vivo application study of xylooligosaccharides

##### Experimental design for studying the effect of carbimazole treatment alone and xylooligosaccharide coadministration on several serum parameters and adrenal histopathology in female rats

Twenty-four female Wistar rats weighing approximately 100–130 g were obtained from the breeding unit of the National Research Centre, Giza, Egypt, for our in vivo study. The rats were given plenty of food and water and were subjected to 12 h. had a cycle of morning and night with constant temperature and humidity. The animals were separated into 4 groups in different cages. Group I received carbimazole (83 mg/kg bw equivalent to 0.02% active ingredient) dissolved in 1% saline in drinking water. Group II received XOS or xylo-oligosaccharides (0.12 g/kg)^33^ coadministered with the drug carbimazole dissolved in 1% saline in drinking water. Group III received 1% saline. Group IV received no supplementation and served as a control.

After three weeks of treatment, the animals were sacrificed by dislocation. Blood was collected from the retro-orbital plexus of the rat eye and subsequently centrifuged at 4000 rpm for 10 min to obtain the serum. The serum was subjected to several biochemical parameters, such as total cholesterol, random glucose and total antioxidant capacity (TOAC), which were determined via colorimetric methods^34,35^. The adrenal glands were histologically evaluated. The glands were separated, cleaned, rinsed in 10% buffered formalin, encased in paraffin and dyed with hematoxylin and eosin (H&E)^36^.

### Statistical analysis

SPSS version 7.5 software (USA) was used to analyze the experimental data. Statistical comparisons between groups were performed by one-way ANOVA followed by post hoc tests.* P* values were considered to be significant at *P* ≤ 0.05.

### Ethical approval

Ethical approval No. 18157 was obtained from the animal ethical committee of the National Research Centre, Giza, Egypt, according to the guidelines of the Helsinki standard ethical laws.

## Results and discussion

### Enzyme activity

After optimization, the *A. terreus* strain RGS Eg-NRC provided the highest xylanase yield (245 U/g). Xylanase, which has a specific activity of 3.9 IU/mg protein, was largely purified when ammonium sulfate was saturated by 60–70%^13^. Given the significance of immobilizing the generated enzyme, the reusability of any enzyme is a critical factor in assessing the sustainability of its industrial application^37^.

### Conditions of the immobilization process of xylanase on alginate/scleroglucan (Alg/Sc.) gel beads

The xylane enzyme was covalently immobilized on alginate/scleroglucan (Alg/Sc) gel beads with an immobilization yield of approximately 53%. By using CCD, the immobilization procedure was enhanced. According to Table 1, loading the enzyme (2 U) onto one gram of activated gel beads and keeping the mixture stationary for 22 h resulted in a nearly 97.2% immobilization yield.

**Table 1:** CCD for optimization of the immobilization process.

Trial	Independent variable		Recovery of immobilized enzyme (%)	Observed immobilization yield (%)	Predicted immobilization yield (%)	Residual
	X1 Time of loading (h)	X2 Loading unit (U/g gel bead)				
1	18 (−1)	2.5 (−1)	0.766	0.55	0.513185	0.036815
2	18 (−1)	3.5 (+ 1)	0.551	0.33	0.271096	0.058904
3	26 (+ 1)	2.5 (−1)	0.92	0.882	0.961569	-0.07957
4	26 (+ 1)	3.5 (+ 1)	0.640	0.349	0.40644	-0.05744
5	14 (−2)	3 (0)	0.532	0.215	0.267832	-0.05283
6	30 (+ 2)	3 (0)	0.981	0.915	0.85156	0.06344
7	22 (0)	2 (−2)	0.995	0.972	0.955778	0.016222
8	22 (0)	4 (+ 2)	0.441	0.153	0.15856	-0.00556
9	22 (0)	3 (0)	0.723	0.53	0.517712	0.012288
10	22 (0)	3 (0)	0.723	0.53	0.517712	0.012288
11	22 (0)	3 (0)	0.70	0.5	0.517712	-0.01771
12	22 (0)	3 (0)	0.72	0.52	0.517712	0.002288
13	22 (0)	3 (0)	0.723	0.53	0.517712	0.012288

The presence of multiple hydroxyl groups in scleroglucan facilitates the attachment and physical coating of polyethylene imine (PEI) onto the beads. The abundant hydroxyl groups promote intermolecular hydrogen bonding, thereby enhancing the stability of the beads in comparison to the alginate beads used alone. Furthermore, this bond augments the effectiveness of immobilization. In addition, this interaction contributes to the mechanical durability of the beads, safeguarding them from degradation or hydrolysis in solution^38–40^.

The loading unit was not significant according to the multiple regression analysis results (Table 2); however, the loading duration had a highly significant effect on the immobilization yield (*P* ˂ 0.05). These results further supported the accuracy of the model since the second-order polynomial model explained 97.1% of the variation in the experimental results, and the R^2^ value of the applied model was 0.971. According to Edwards et al.^41^ the R^2^ value (> 0.9) of the applied model is correct.

**Table 2:** Analysis of CCD.

	Coefficients	Standard error	t stat	*p*-value
Intercept	−0.99905	0.663842	−1.50495	0.147966
X1	0.125009	0.031665	3.947866	0.000795
X2	0.225509	0.249465	0.903968	0.376773
X12	0.000656	0.000436	1.502415	0.148615
X22	0.039457	0.027926	1.412894	0.173061
X1X2	−0.03913	0.008355	−4.6829	0.000143

The high F value (136.1138) and extremely low *P* value (9.81E−15) in the analysis of variance (ANOVA) results reflect the statistical significance of the model terms utilized in the current investigation. The following second-order polynomial equation was produced from the regression analysis of the experimental results and was used to compute the expected immobilization yield:

$${\varvec{Y}} = - 99905 + 0.125009{\varvec{X}}_{1} + 0.225509 X_{2} + 0.000656 {\varvec{X}}_{1}^{2} + 0.039457{\varvec{X}}_{2}^{2} - 0.0391{\varvec{X}}_{1} {\varvec{X}}_{2}$$

**Figure 2:**
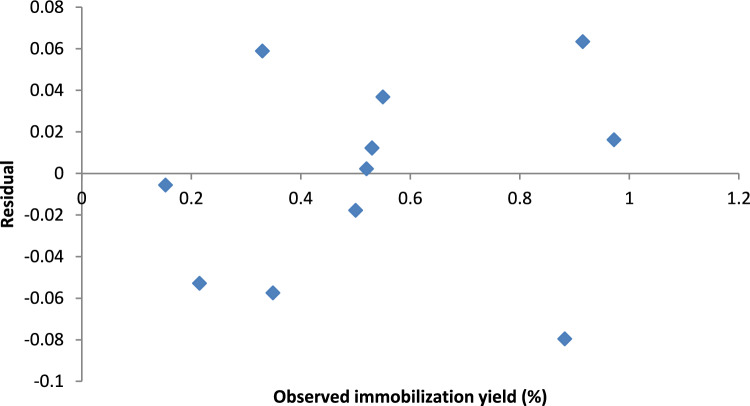
Residual plot.

Additionally, residual analysis (Fig. 2), which was developed by plotting the residuals (the observed-predicted values) against the immobilization yield (the response), showed that the residuals were consistently distributed throughout the range and fell in a symmetrical pattern, confirming that the model is accurate for the majority of the observed results.

### Characterization of the modified gel beads using Fourier transform infrared (FT-IR) spectroscopy

**Figure 3:**
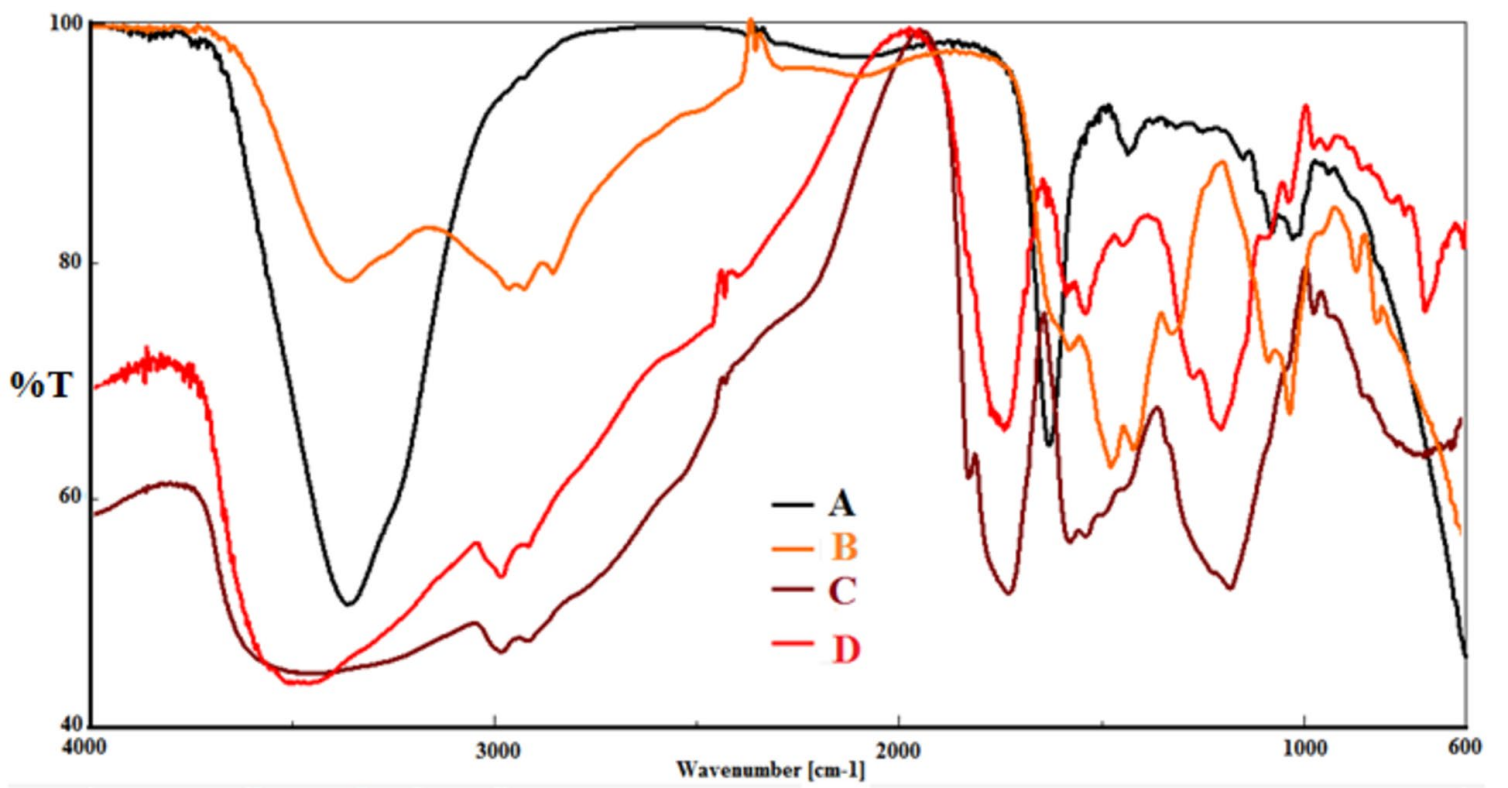
**3**FT-IR spectra of alginate/scleroglucan (**A**), alginate/scleroglucan/PEI (**B**), alginate/scleroglucan/PEI/GA (**C**) and alginate/scleroglucan/PEI/GA/enzyme (**D**).

The FT-IR spectroscopy results for the four formulations (alginate/scleroglucan, alginate/scleroglucan/PEI, alginate/scleroglucan/PEI/GA, and alginate/scleroglucan/PEI/GA/enzyme) are shown in Fig. 3. According to curve A relating to the mixture of alginate and scleroglucan, an identifiable band at 3360 cm^−1^ was observed that is related to OH and found naturally in the structure of alginate and scleroglucan, as was a band at 1638 cm^−1^ that is related to the carbonyl group (C=O) of scleroglucan. The interaction between the carbonyl groups in the mixture and the amine group (NH_2_) on the surface of the alginate/scleroglucan gel beads results in a new band at 1478 cm^−1^ that is related to CN groups, as shown in Curve B, which is related to aminated alginate/scleroglucan gel beads. In curve C, which corresponds to activated alginate/scleroglucan gel beads, a band corresponding to amines is visible at wavelengths between 3120 and 3622 cm^−1^. In addition to the band for CN groups at 1451 cm^−1^, this band corresponds to the CN group, which is the result of the interaction between the carbonyl groups of glutaraldehyde and the amine groups on the surface of the gel beads. The band at 1611 cm^−1^ is specific to the terminal carbonyl groups of glutaraldehyde. On the other hand, as shown in curve D, when the enzyme and activated gel beads reacted, the various amine groups in the enzyme caused the amine range to expand and reach 3271–3594 cm^−1^. There is also a band at 1516 cm^-1^ that pertains to CN groups as a result of the interaction between the carbonyl groups on the surface of activated alginate/scleroglucan gel beads and the amine group (NH_2_) on the surface of enzymes^4,29^.

### Stability and operational conditions of the immobilized xylanase

**Figure 4:**
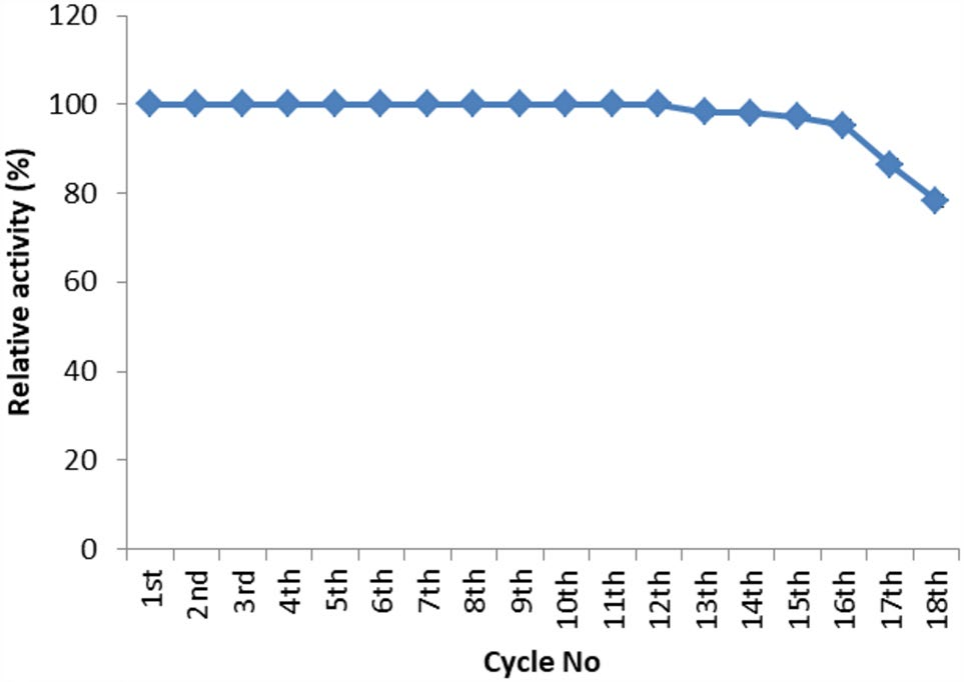
Operational stability of xylanase immobilized onto alginate/scleroglucan gel beads.

The operational stability, also known as the storage stability and repeated use stability, determines how reusable an enzyme is. The immobilized enzyme maintained its full activity for more than 12 cycles, reaching 78.33 after 18 cycles, according to the findings of repeated use (Fig. 4). The substrate might be the reason for this decline in enzyme activity becoming toxic after repeated usage of the enzyme^42^.

From an economic perspective, this is a major discovery because it will lower the overall cost of the enzyme. Additionally, the immobilized enzyme was allowed to react for more than 41 days at 4 °C, in contrast to the free enzyme, which has stable activity for 8 days and had to be contaminated, at which point the enzyme was fully active before it was removed. The immobilized enzyme is appropriate for extensive industrial application due to its great operational stability. According to Mostafa et al*.*^28^ the operational stability of xylanase immobilized on (Alg + PEI/Na +) gel beads has increased. For instance, after eight consecutive reactions, 96% of the initial activity of xylanase was retained after it was immobilized on (Alg + PEI/Na +) gel beads. However, following sixteen cycles, there was a slight and gradual decrease in enzyme activity, which eventually reached 73% of its initial level.

### Descriptions of xylanase in the immobilized state

#### pH profile of the immobilized xylanase

**Figure 5:**
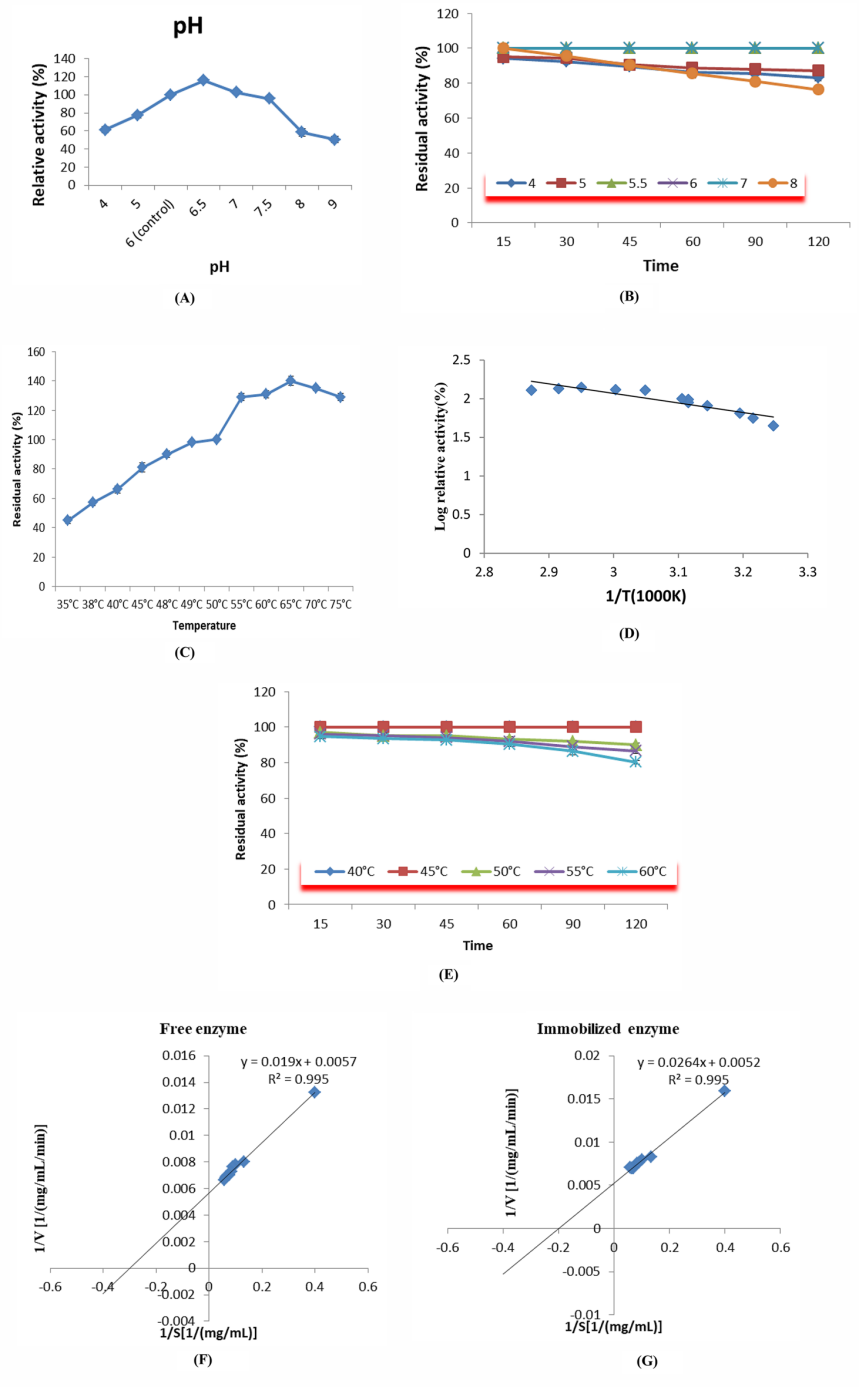
Effect of the reaction pH (control is pH 6) (**A**), preincubation at different pH values for different time intervals (the activity of the enzyme without preincubation was considered 100% activity), on the activity of the immobilized enzyme (**B**), the reaction temperature (50 °C is the control) (**C**), the activation energy (Ea) (**D**), and preincubation at different temperatures for different time intervals (the activity of the enzyme without preincubation was considered 100% activity) on the activity of the immobilized enzyme (**E**). Lineweaver–Burk plots were used to determine the values of the Michaelis–Menten constant (k_*m*_) and maximum reaction rate (Vmax) for both free (**F**) and immobilized xylanase enzymes (**G**).

When 0.05 M phosphate buffer was utilized, the immobilization of xylanase on (Alg/Sc.) Gel beads resulted in a minor shift in the reaction’s optimum pH (6.5), although studies of the enzyme’s activity as a function of reaction pH (Fig. 5A)^18^ showed that the maximum pH of 6.0 was reached by free xylanase. The structural and functional stability of the immobilized xylanase (Alg/Sc.) can be used to explain these results. The presence of gel beads is predominantly influenced by the composition and structure of the carrier as well as the structure of the enzyme, and both of these factors significantly influence the enzyme's catalytic activity following immobilization^43^. Additionally, for up to two hours, the immobilized xylanase maintained 100% of its enzyme activity (Fig. 5B). The maximum activity of xylanase that was immobilized under moderately neutral conditions was comparable to that reported by Wong et al*.*, who reported that xylanase immobilized on metal ions had an optimum pH of 7.0^44^.

#### Immobilized xylanase temperature profile

One of the most important features of immobilization technology is the reaction temperature. The immobilized xylanase functioned best at 65 °C, while the optimum temperature was 55 °C, as reported previously^18^. The enzyme activity decreased after that point, possibly because of the thermal denaturation of the enzyme (Fig. 5C).

The need for greater activation energy in the case of immobilized enzymes than in the case of free enzymes can explain the difference in the optimal reaction temperature for immobilized xylanase. The structural flexibility of the enzyme within the gel microenvironment may vary as a result of these modifications^45–49^.

#### Arrhenius plot of the immobilized enzyme

The Arrhenius equation can be used to calculate the activation energy and efficiency of chemical processes. When a reaction obeyed the Arrhenius equation, it showed a linear relationship with the log of residual activity vs. time. This indicated the enzyme's first-order kinetic process, from which the activation energy (Ea) may be computed using the gradient and intercept. The graph of the amount of immobilized xylanase produced and this equation were identical.

Arrhenius plots were then used to calculate the activation energy (Ea) of the catalyst for *A. terreus* xylanase (Fig. 5D). For the xylanase Arrhenius plots, the regression formula was

$$y = 1.2359x + 5.776$$

The results showed that the Ea of the immobilized xylanase was 10.049 kJmol, which is less than that of the free enzyme (23.919 kJmol). This finding was previously reported^18^. With decreasing Ea, less energy is needed to reach the active site of the enzyme–substrate complex. Immobilized xylanase is more suitable for industrial applications due to these characteristics and its low activation energy, which decreases the overall manufacturing price.

#### Temperature stability

The enzyme exhibited no decrease in activity after 120 min of incubation at 40 °C or 45 °C, as shown in Fig. 5E. However, after 120 min at 50 °C, 90% of the activity remained, and at 60 °C, the immobilized enzyme could only sustain 80.22% of its activity, as opposed to its free enzyme, which only managed to maintain approximately 72.55% of its activity^18^**.**

#### Effect of different substrate concentrations

The activity of both the immobilized and free enzymes at various substrate concentrations (xylan) was plotted using a Lineweaver‒Burk plot. The Michaelis‒Menten constants (K_*m*_) and maximal activity (V_*max*_) were computed from this graph. The K_*m*_ of the free enzyme was 3.57 mg ml^−1^, while the k*m* of the immobilized enzyme was 5.0 mg ml^−1^. Furthermore, as shown in Fig. 5F and G, the V_*max*_ of the free and enzymes immobilized were 172.34 and 200 mg ml^−1^ min^−1^, respectively. Table 3 shows a comparison of *Aspergillus terreus* xylanase properties after immobilization by different polymers.

**Table 3:** Comparison of *Aspergillus terreus* xylanase properties after immobilization by different polymers.

Polymer	Properties	Refs.
Alginate-polyethyleneimine/Na +	pH (5.5),Temp (60 °C), Km (13.33 mg/mL), Vmax (7.463 Umin^-1^), Reusability (80% (16)^a^)	^25^
Protein-inorganic hybrid nanoflower	pH (5.5),Temp (70 °C), Km (1.60 mg/ml), Vmax (455 μmol/min/mg), Reusability (75.8 (10)^a^)	^42^
Aminated superparamagnetic graphene oxide nanocomposite	pH (7.5),Temp (70 °C), Km (80.0 mM), Vmax (2.5 mM/min), Reusability (50% (4)^a^)	^43^
Gellan gum-agar beads	pH (5–8),Temp (50–70 °C), Km (5.55 mg mL^-1^), Reusability (85.7% (11)^a^)	^44^
Microbial Scleroglucan	pH (6.5),Temp (65 °C), Km (4.35 mg ml^−1^), Vmax (200 mg ml^−1^ min^−1^), Reusability (78.33% (18)^a^)	This study

^a^The number of cycles.

### Hydrolytic conditions for the production of xylooligosaccharides

In 250-mL screw-capped bottles with 100 mL of 2% xylan (in 0.05 M phosphate buffer at pH 5.8) and immobilized enzyme (5 U/ml) and incubated at 40 °C for two hours, the ability of the resulting enzyme to break down the wood xylan polymer was assessed. The polymer was then collected, cleaned with buffer, and used again to make xylooligosaccharides. Next, the clear supernatant was centrifuged at 7000 rpm for 10 min (4 °C). Using DNS, the quantities of reducing sugars in the hydrolysis products were determined. Therefore, using the same solvent system and spraying agent as^32^, thin layer chromatography was used to identify the hydrolysis product patterns and the reusability of the immobilized enzyme (Fig. S2).

**Figure 6:**
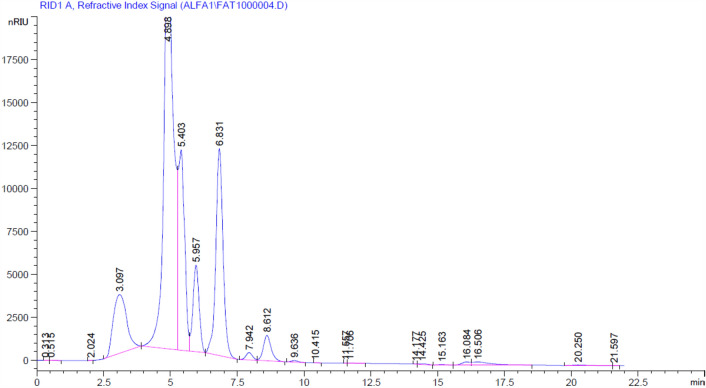
HPLC analysis of beech wood xylan hydrolysate.

Figure 6 shows the HPLC analysis of the dried hydrolysate obtained from the reaction and enzymatic hydrolysis of beech wood xylan. The primary products were found to be xylose, xylobiose, and xylotriose, according to the results. According to the analysis, their relative retention times were 8.61, 7.94, and 6.83 min, respectively. The xylan hydrolysate contained xylose (3.98 mg/g), xylobiose (143.49 mg/g), and xylotriose (256 mg/g). These results were in agreement with those of Wahba et al.^50^ who reported that the immobilized enzyme had the capacity to combine xylooligosaccharides with a modest amount of xylose. Xylooligosaccharides have many biological activities, including antioxidant^18^ and prebiotic activities^51^.

### In vivo study of xylooligosaccharides

#### Biochemical studies of the effects of carbimazole alone and xylooligosaccharide coadministration on several serum parameters in female rats

The antithyroid drug carbimazole is one of the most popular therapeutic agents used by physicians worldwide for the treatment of hyperthyroidism^52^. This disease is characterized by the overproduction of thyroid hormones (T4 and T3) and is associated with many enzymatic and hormonal disorders^35^. Although carbimazole successfully decreases serum thyroid hormone levels, it may strongly affect the adrenal glands and other organs^53–57^. The drug can also lead to the initiation of hypothyroidism due to a reduction in thyroid hormone levels, which in turn helps in the gradual increase in obesity ^58^.

As Table 4 illustrates, the data used in this investigation are consistent with earlier findings. Only the serum glucose and cholesterol levels were significantly greater in the carbimazole-treated group than in the control group. This group also showed a significant decrease in the overall antioxidant capacity of the serum. Conversely, however, significant improvements in the serum cholesterol concentration and total antioxidant capacity were observed in the XOS coadministered with carbimazole group (treated group), in addition to the serum glucose level showing no discernible change, as indicated by Table 4.

**Table 4:** Effect of Carbimazole drug only and xylooligosacarides co-administration on some serum parameters in female rats.

Groups parameters	Carbimazole administrated (Positive)	Co-administration of XOS and carbimazole (Treated)	1% Saline control	Control normal
Total cholesterol (mg/dl)	125 ± 7.10^a,c,d^	97.23 ± 7.41^a,b,c^	74.58 ± 5.42^b,d^	64.19 ± 0.38^b,d^
Random glucose (mg/dl)	91.66 ± 0.01^c^	83.33 ± 8.33	97.21 ± 7.98^c^	75.78 ± 18.51^a,d^
TOAC (mM/L)	0.0033 ± 0.015^c,d^	0.02 ± 0.001b	0.05 ± 0.001^c^	0.024 ± 0.0071^a,b^

*P*^*a*^ significant to 1% saline gp.

*P*^*b*^ significant to positive gp.

*P*^*c*^ significant to control normal.

*P*^*d*^ significant to treated group.

#### Histopathological examination of adrenal gland-only and xylooligosaccharide coadministration

##### Adrenal cortex examination

**Figure 7:**
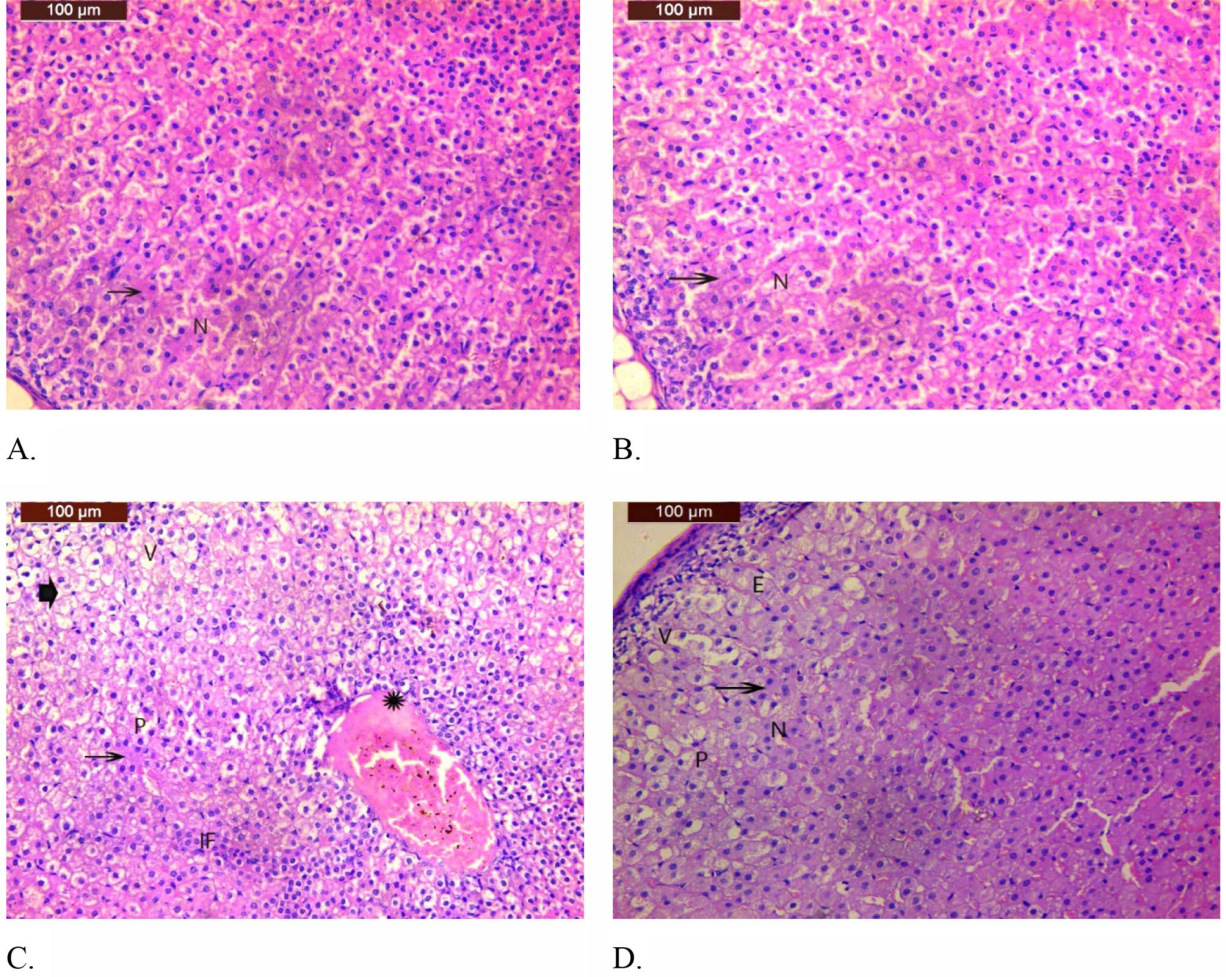
(**A**) Photomicrographs of adrenal gland sections from the control group were normal in structure. The cortex layer consists of three zones with oval to rounded nuclei (N) and acidophilic cytoplasm (arrow). (**B**) A photomicrograph of a section of the adrenal gland from the saline treatment group showed that the structure was normal. The cortex layer consists of three zones with oval to rounded nuclei (N) and acidophilic cytoplasm (arrow). (**C**) Photomicrograph of a section of the adrenal gland of a rat treated with carbimazole showing degeneration, necrosis (arrowhead), pyknosis (P), and acidophilic cytoplasm (arrow); cytoplasmic vacuolation in the cells of the zona glomerulosa and zona fasciculata (V); congestion of blood vessels (star); and inflammatory cell aggregation (IF). (**D**) Photomicrographs of adrenal gland sections from rats treated with carbimazole and XOS showing that most of the morphological changes observed in the positive control group were improved, with acidophilic cytoplasm (arrow), slight cytoplasmic vacuolation (V), pyknosis (P), and sinusoids in the zona reticularis filled with erythrocytes in the cells of the zona glomerulosa and zonafasciculata (**E**).

The anatomy of the adrenal glands in both the control and saline groups seemed normal. The zona glomerulosa, zona fasciculata, and zona reticularis are the three zones that make up the cortex layer. The zone had oval to rounded nuclei and acidophilic cytoplasm (Fig. 7A,B).

Histopathological examination of adrenal gland tissues from rats treated with carbimaziole revealed degeneration, necrosis, pyknosis, and acidophilic cytoplasm, in addition to cytoplasmic vacuolation in the cells of the zona glomerulosa and zona fasciculata. Additionally, congestion of blood vessels and inflammatory cell aggregation were observed (Fig. 7C).

Examination of sections from the groups treated with carbimazole and XOS revealed that most of the morphological changes observed in the positive control group improved. However, the acidophilic cytoplasm, slight cytoplasmic vacuolation, pyknosis, and sinusoids in the zona reticularis were filled with erythrocytes from the cells of the zona glomerulosa and zona fasciculata (Fig. 7D).

##### Adrenal medulla examination

**Figure 8:**
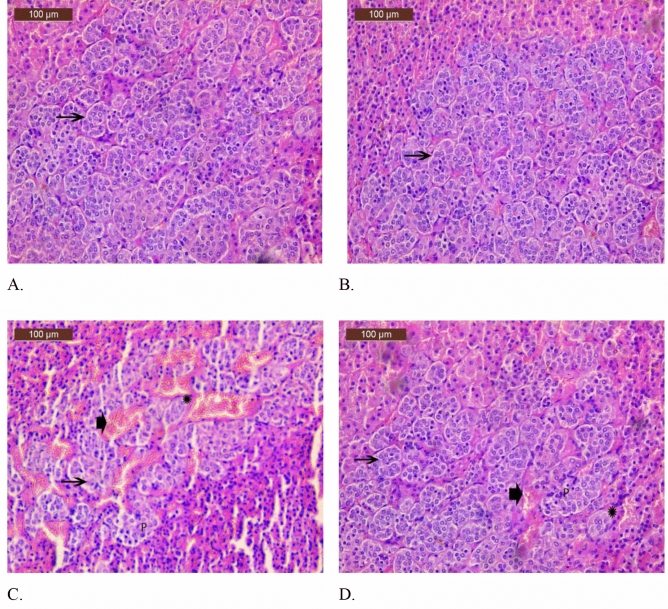
(**A**) Photomicrograph of the adrenal medulla of a rat in the control group showing normal architecture of the adrenal gland. Polyhedral chromaffin cells (arrows). (**B**) Photomicrograph of the adrenal medulla of a rat in the saline group showing normal architecture of the adrenal gland. Polyhedral chromaffin cells (arrows). (**C**) Photomicrograph of the adrenal medulla of rats in the carbimazole-treated group showing loss of normal architecture (arrow), large foci of necrotic chromaffin cells (star), vascular congestion (arrowhead), and pyknotic nuclei (P). (**D**) A photomicrograph of the adrenal medulla of rats treated with carbimazole and XOS showing similar histopathological changes to those of the control group in addition to a few foci of necrotic chromaffin cells (star) and mild vascular congestion (arrowhead) with few pyknotic nuclei (P).

In the control and saline groups, chromaffin cells appeared as large polyhedral cells clustered in cords and clusters in the adrenal medulla slices and displayed normal characteristics. Blood vessels divide these cords and clusters (Fig. 8A,B). Staining of the adrenal medulla from rats given carbimazole treatment revealed pyknotic nuclei, extensive clusters of necrotic chromaffin cells, loss of normal architecture, and vascular congestion (Fig. 8C). Minor vascular congestion is present along with a few foci of necrotic chromaffin cells and a few pyknotic nuclei, and the stained sections of the adrenal medulla from rats treated with carbimazole and XOS showed nearly identical histological changes to those of the control group (Fig. 8D).

In the rat group that received only carbimazole, as shown in the Figs. 7C and 8C, there were clearly visible structural alterations in both the cortex and the medulla of the adrenal gland as opposed to those in the control group (7A–D).

Sánchez et al.^59^ and Skórka-Majewicz et al.^60^ reported that a normal structure of the adrenal cortex was observed after 24 h of injection into mice treated with saline; on the other hand, injecting snake venom altered the structure of the mouse adrenal cortex. In addition to chemicals, natural or synthetic toxins can affect the efficiency of the adrenal cortex and adrenal hormone secretions. Tseilikman et al.^61^ reported that stress leads to alterations in adrenal gland functions. Recent studies have shown that the morphological alterations observed in adrenal gland tissues associated with alterations in secreted hormones can translate to adapt from the gland to resist certain external stresses (this may occur for a distinct duration) ^62–64^. Numerous studies have reported on the outstanding regeneration ability of adrenal gland cells and their ability to adapt to specific physiological and pathological conditions^65–67^ These authors attributed this ability to the crosstalk between the different cell types of the gland and their progenitor cells and to vascularization support, which in turn co-operates to create a suitable microenvironment surrounding the gland. Kanczkowski et al.^68^ revealed the relationship between the progression of many diseases in the adrenal gland and alterations in the microenvironment surrounding the gland.

In the present study, the adrenal gland experienced two main stresses. The first was recognizing the medication as a chemical toxin, and the second was the development of fat and a corresponding decrease in total antioxidant capacity. Increasing oxidative stress, which is linked to the onset of obesity, is always reflected in declining antioxidant capacity^69^.

The coadministration of xylo-oligosaccharides with the medicine carbimazole was suggested for the first time in the present study to reduce pharmacological problems that can damage the adrenal gland. Compared with those in the control group, the cortex and medulla of the adrenal tissues in the treated group showed morphological improvement, as shown in the Figs. 7D and 8D, and in the group that received carbimazole, as demonstrated in the Figs. 7C and 8C.

These results paralleled the significant improvements observed in the serum cholesterol and TOAC levels in the treated group, as shown in Table 4. These data reflected the antioxidant influence and hypolipidemic effect of XOS in alleviating carbimazole complications that harm the adrenal gland in addition to helping to accelerate regeneration in the short term. These results are consistent with those of Li et al*.*^70^ and Boyanov et al*.*^71^, who confirmed the antioxidant and hypolipidemic effects of XOS. We investigated the harmful effects of CBZ on the liver, kidneys, thyroid^66^ and, ultimately, adrenal glands.

Most of the studies discussing the effect of carbimazole on adrenal gland morphology have not proposed any solutions or medications to mitigate the harmful effects of the drug on the glands. This research primarily focused on the drawbacks of CBZ administration to various organs, particularly the thyroid gland, and rarely mentioned the adrenal glands.

Based on our previous work, coadministration of XOS with CBZ can enhance the efficacy of CBZ in both the thyroid gland and the adrenal gland. This indirectly suggests the correction of signals between the brain–thyroid axis and the brain–adrenal axis, ensuring the safe coadministration of XOS with the drug in cases of disease^72^.

## Conclusion

The xylanase derived from *Aspergillus terreus* is effectively immobilized by utilizing a complex of microbial Scleroglucan and alginate polymer. This particular enzyme can degrade xylan, a polysaccharide predominantly present in beechwood, yielding xylo-oligosaccharides. The novel approach of combining these xylo-oligosaccharides with carbimazole demonstrates a potential avenue for mitigating the deleterious impact on the adrenal glands. This application of xylo-oligosaccharides is pioneering in nature. Furthermore, the use of XOS has been shown to reduce oxidative stress associated with obesity while concurrently promoting the rejuvenation of adrenal gland cells.

### Supplementary Information

Supplementary Information.

## Data Availability

All the data generated or analyzed during this study are included in this published article and its supplementary information file.
