# Supplementary material for: Co-administration of xylo-oligosaccharides produced by immobilized Aspergillus terreus xylanase with carbimazole to mitigate its adverse effects on the adrenal gland
Source: Sci Rep. 2024 Jul 30;14:17481. doi: 10.1038/s41598-024-67310-4 (PMC11289116; doi:10.1038/s41598-024-67310-4)
Supplement: Supplementary file 1 — Supplementary Information. [file 41598_2024_67310_MOESM1_ESM.docx]

**Co-administration of xylo-oligosaccharides produced by immobilized *Aspergillus terreus* xylanase with Carbimazole to mitigate its adverse effects on the adrenal gland.**

Shaimaa A. Nour^a^, Doaa S. Foda^b^, Islam A. Elsehemy^a^, Mohamed E. Hassan^a,c,*^

**^a^** Chemistry of Natural and Microbial Products Department, Pharmaceutical and Drug Industries Research Institute, National Research Centre, El Behouth Street, Cairo 12622, Egypt.

**^b^** Therapeutic Chemistry Department, Pharmaceutical and Drug Industries Research Institute, National Research Centre, El Behouth Street, Cairo 12622, Egypt.

**^c^** Centre of Excellence, Encapsulation and Nano Biotechnology Group, Chemistry of Natural and Microbial Products Department, Pharmaceutical and Drug Industries Research Institute, National Research Centre, El Behouth Street, Cairo 12622, Egypt.

*** Corresponding Authors:** Mohamed E. Hassan ([mohassan81@gmail.com](mailto:mohassan81@gmail.com))


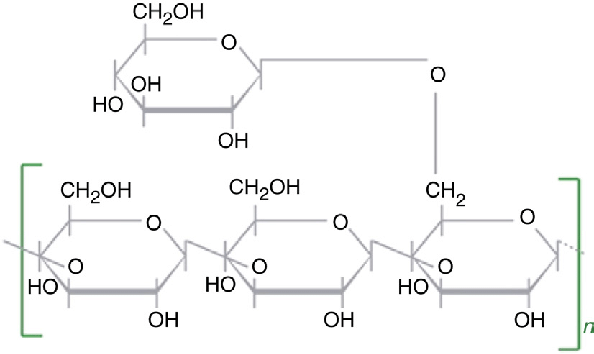


S1 Chemical Structure of Scleroglucan


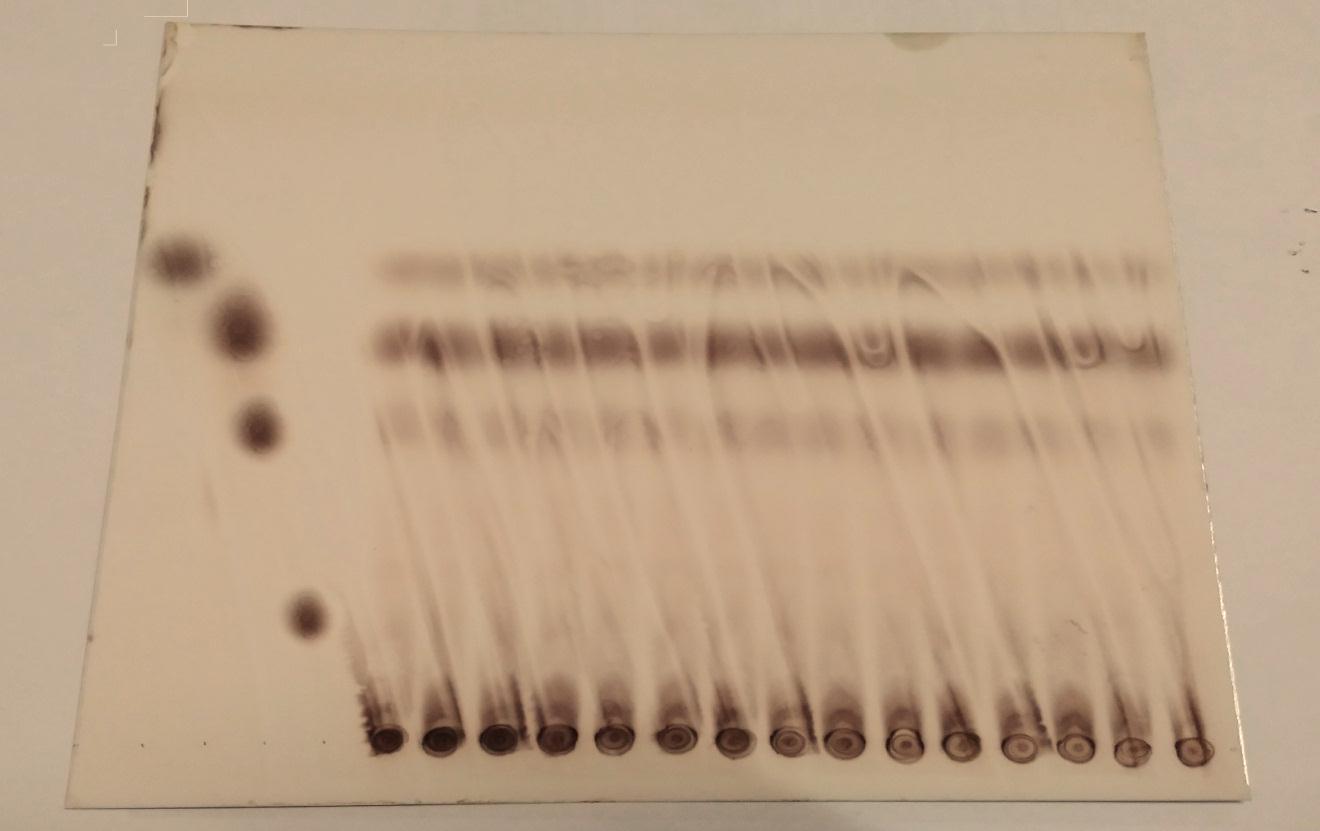


X1 X2 X3 X4 ( 1 2 3 4 5 6 7 8 9 10 11 12 13 14 15) Cycle

S2: TLC plate of hydrolysis product of xylan by immobilized *Aspergillusterreus*xylanase at 15 cycles .X1: mono, X2: Di, X3: Tri, X4: Tetra, lan 1: 1^st^ cycle, lan 2: 2^nd^ cycle, and from lan 3to lan14 are from 3^rd^ cycle to15^th^ cycle.
